# Symbolic Understanding and Word–Picture–Referent Mapping from iPads in Autism Spectrum Condition: The Roles of Iconicity and Engagement

**DOI:** 10.1007/s10803-020-04404-8

**Published:** 2020-02-08

**Authors:** Bethany R. Wainwright, Melissa L. Allen, Kate Cain

**Affiliations:** 1 Department of Psychology, Lancaster University, Lancaster, LA1 4YF UK; 2 School of Education, University of Bristol, Bristol, UK

**Keywords:** Symbolic understanding, Word–picture–referent mapping, Autism, iPad, Engagement

## Abstract

We investigated symbolic understanding, word–picture–referent mapping, and engagement in children with autism spectrum condition (ASC) and ability-matched typically developing children. Participants viewed coloured pictorial symbols of a novel object (given a novel name) on an iPad in one of three conditions: static 2D images and either automatically or manually rotating images (providing a three-dimensional context). We found no significant difference in word–picture–referent mapping between groups and conditions, however, children who manually rotated the picture had greater on-screen looking time compared to other conditions. Greater visual attention related to more successful word–picture–referent mapping only for the children with ASC. Interactive iPad tasks may increase visual attention in both typical and atypical populations and greater visual attention may benefit word–picture–referent mapping in ASC.

Communication problems are one of the main reported weaknesses associated with Autism Spectrum Condition (ASC) (Alzrayer et al. 2014; American Psychiatric Association 2013; Caruana et al. 2017; Paul et al. 2017). For children across the spectrum, receptive and expressive language development can be significantly delayed (Anderson et al. 2007; Wodka et al. 2013). Children with ASC often communicate using pictorial symbols as an alternative to speech (Bondy and Frost 1994; Kasari and Patterson 2012; Lord and Jones 2013) and demonstrate a relative strength in visuo-spatial processing compared to language (Kumar 2013), yet knowledge regarding how children with ASC understand pictures on a symbolic level is relatively scarce. Critically, existing research suggests differential learning mechanisms are in place for chidren with ASC and TD children (Hartley and Allen 2014b; 2015a, b; Preissler 2008).

Symbolic understanding of word–picture–referent relations emerges at around 18–24 months in typically developing (TD) children (Ganea et al. 2009; Preissler and Carey 2004). Word–picture–referent relations is here defined as the knowledge that a label refers to both the pictorial symbol and the real-world referent it depicts (Hartley and Allen 2014b, 2015a, b). Children in their second year of life can successfully fast-map new nouns to their intended referents immediately after label exposure (Munro et al. 2012) and retain the new noun over short time periods after a single instance of labelling (Spiegel and Halberda 2011). At 24 months, children demonstrate a shape-bias in object categorisation (Samuelson and Smith 1999), generalising the mapping of a new noun from the original referent to a differently coloured referent of the same shape (Hartley and Allen 2014a). However, children with ASC often have specific difficulties understanding that words and pictures symbolically refer to objects (Hartley and Allen 2014b; Preissler 2008). Instead, they show associative mapping of word–picture–referent relations, restricting a label to the symbol itself and failing to generalise to a real-world referent or differently coloured examplars. This is in contrast to the referential mapping exhibited by TD children, who readily generalise a label given to a picture to its corresponding object (Preissler and Carey 2004). The differences in word–picture–referent mapping mechanisms between children with ASC and TD children could have a significant impact on word acquisition and the flexible use of language for children with ASC.

Increasing the iconicity of pictorial symbols has been found to be an effective way of improving the referential understanding of children with ASC (Hartley and Allen 2015a). Iconicity is the extent to which an image visually resembles its referent (Sirota et al. 2014). Images can vary in visual iconicity, with printed words defined as opaque, black and white images defined as translucent, and coloured photographs defined as transparent (Fuller et al. 1997). Ganea et al. (2008) investigated whether the visual iconicity of an image in a printed picture book influences the extent to which TD 15–18 month-old infants generalised the label of a picture to a real-world referent. Infants more often generalised the label to the real-world referent when the pictures were realistic and transparent (colour photographs) than when they were less realistic and translucent (cartoons). This was especially apparent for the 15-month-old infants. The researchers concluded that increasing the iconicity of pictures is beneficial in picture books because it enhances symbolic understanding, especially for younger infants.

As stated above, evidence suggests that children with ASC may interpret symbols, specifically pictures, in a different way to TD children (Hartley and Allen 2015b; Preissler 2008). Hartley and Allen (2014b) compared children with ASC (*M*_*age*_ = 9.7 years) and TD 2 to 5-year-olds in their ability to match abstract and iconic pictures with their intended referents. Children with ASC relied highly on visual resemblance and matched pictures to their referents more often with iconic than abstract images. In contrast, the TD children successfully matched both types of pictures with their intended referents. Their findings suggest that low-functioning children with ASC rely on resemblance, and do not take the intention of the artist into account, whereas TD children can understand the intention of the artist even in the absence of high visual resemblance. Thus, it appears that, unlike TD children, children with ASC rely on a high level of iconicity when matching a picture to a referent.

As another test of iconicity, Hartley and Allen (2015b) presented children with ASC (*M*_*age*_ = 9.7 years) and TD 2 to 5-year-olds matched on receptive vocabulary score with pictures of novel objects that varied in iconicity from grey and coloured line drawings to black and white and coloured photographs. For each trial, a novel word was paired with a novel picture. In a ‘mapping’ test, participants were asked to select the named item from a choice of the picture shown in the training phase and the previously unseen referent object. In a subsequent ‘generalisation’ test, the object was replaced with a differently coloured version of the same object and participants were again asked to indicate the referent. The TD children selected the object in the mapping and generalisation tests in the majority of trials, regardless of condition. In contrast, children with ASC often selected the picture they had been shown, suggesting that they had formed an association between the word and the picture and failed to generalise the word to the object. However, children were more likely to choose the object in both the mapping and generalisation tests as iconicity increased, with the fewest object selections for the black and white line drawings and the most for the colour photographs. This indicates that iconicity supports symbolic understanding.

As noted, symbols are essential to support the flexible use of language for those with communication difficulties, such as children with ASC. In recent years, the Apple iPad has become increasingly popular as a learning aid for students (Geer et al. 2016; Neumann 2018), with a wide variety of educational applications available (Alzrayer et al. 2014). The portable and robust nature of iPads and tablets, combined with the media capabilities and applications on offer (Banister 2010) make it an appealing alternative to paper-based learning for teachers in both specialist (Cardon 2012; Kagohara et al. 2013; King et al. 2017) and mainstream education (Gitsaki and Robby 2015). iPad-based learning has been found to increase student engagement and reduce problem behaviour in both typically and atypically developing populations (El Zein et al. 2016; Kucirkova et al. 2014). Moreover, touch-screen interactivity allows for more information to be conveyed to the child through touch and motion and for information to be processed as an active experience, which may change how the information is encoded and stored (Russo-Johnson et al. 2017).

The educational value of interactive touch-screen learning is very much in debate (Kirkorian 2018). Interactivity may increase the cognitive load of young children more-so than non-interactive material, impeding learning. However, it may also increase user-engagement and guide visual attention towards relevant features, improving learning. Indeed, studies to date report both positive (Highfield and Goodwin 2013; Schwartz and Plass 2014; Xie et al. 2018) and negative (Radesky et al. 2015; Russo-Johnson et al. 2017) influences of interactivity on learning. Highfield and Goodwin (2013) stated that interactive iPad learning (through touch, repetition and exploration) complements the preferred learning style of those in infancy and early childhood. One claim is that iPads foster more active involvement for young children, rather than passively listening to information in the classroom (Kucirkova 2014). Relevant here is work examining interactive e-books and applications as a learning aid for young pupils. On-screen interactivity increases language learning and reading skills in young children; one possible mechanism is that touch-screens provide real-time feedback to children and appropriately timed responses which are more engaging and similar to real-life interactions (Radesky et al. 2015). A recent meta-analysis of 36 studies found that young children learn a wide range of materials better from touch-screen devices than non-touch screen media (Xie et al. 2018).

However, there is evidence that the interactivity offered by e-books may be a potential hindrance to learning (Krcmar and Cingel 2014). Krcmar and Cingel found that preschoolers showed greater learning from traditional books compared to e-books, with more relevant discourse between parents and children when sharing traditional print books. Moreover, Russo-Johnson et al. (2017) found no difference in young children’s word learning when images were viewed passively compared to an interactive condition.

Despite mixed results, iPads and touch-screen technology have been widely credited with increasing the engagement of learners in both mainstream and specialist education, with many reporting a user-preference towards iPads at the expense of traditional paper-based alternatives (Richter and Courage 2017). However, very little research has defined engagement into measurable categories and, critically, examined the relationship *between* engagement and learning outcomes. One exception is a study by Richter and Courage (2017) that compared pre-schooler engagement in an e-book and a traditional book in terms of various categories including visual attention (looking time at the book/screen, adult and off-book/screen), communication (such as labelling and speech relevant to the story), and ‘persistence, enthusiasm and compliance.’ Measures of these different types of engagement during a storybook task were examined in relation to comprehension of the book. Results showed greater on-task looking time for the e-book compared to the traditional book and higher persistence, enthusiasm and compliance. Low levels of communication were reported across both conditions, which the authors note may be due to the young age of the participants. Despite higher engagement in the e-book condition, storybook comprehension did not differ between conditions. The researchers concluded that interactive iPad applications may be beneficial for engaging and motivating learners, however they may not influence learning.

Despite much interest surrounding the use of the iPad as an educational tool for children with ASC (Cardon 2012; Chmiliar 2017; Kagohara et al. 2013; Whitehouse et al. 2017), most research has focussed on TD populations and the effects of interactivity on symbolic understanding is yet to be investigated in both typical and atypical development. The overall efficacy of word–picture–referent mapping via iPads is very much in debate and remains an open and essential question (Allen et al. 2016).

Presenting stimuli on a screen has the potential to enhance the iconicity of an image beyond traditional picture books, by providing the three-dimensional context of a real-world object. As a higher level of iconicity has been found to increase symbolic understanding (Allen et al. 2016; Hartley and Allen 2015b), providing three-dimensional context to images may lead to more successful word mapping. Moreover, the iPad touch-screen allows for interactivity and manual exploration of pictorial symbols. When participants touch and interact with images on an iPad screen, they may process the information more deeply or actively (Russo-Johnson et al. 2017), which may benefit the mapping of new symbols. The interactivity provided by the touch and motion may lead to greater engagement (such as visual attention and communication) in the task compared to non-interactive conditions (Radesky et al. 2015; Richter and Courage 2017), which may positively impact subsequent word mapping.

The current study contrasts the word–picture–referent mapping and symbolic understanding of children with ASC and TD controls from images displayed on an iPad. Children completed a training phase in which pictorial symbols of unfamiliar objects were presented on an iPad paired with an unfamiliar spoken label. A critical contrast was whether the image was displayed as a static 2D image (similar to a printed photograph) or as a 3D image. For the 3D images, participants could view either the image rotating (automatic condition) or could rotate the images themselves by touching the screen (interactive condition). Children were then immediately tested on their word–picture–referent mapping. Studies have demonstrated that children can perform accurately on immediate mapping tests despite having poor retention after a delay (Horst and Samuelson 2008). Therefore, children were tested again after two-weeks in a subsequent retention test. Children were also video-recorded during the training phase to examine the relationship between engagement and successful symbolic mapping. Engagement categories were adapted from the coding scheme proposed by Richter and Courage (2017) and included visual attention (screen looking, adult looking and off-screen (environment) looking) and communication (labelling and relevant speech) as measures of engagement.^1^

The first aim was to determine whether symbolic responding and label generalisation will differ by group (ASC vs TD) and condition (3D images vs 2D images). The second aim was to determine if engagement (visual attention and communication) varies by group and/or condition. The third aim was to examine whether higher engagement is contingent with both immediate symbolic mapping and retention after a delay.

It is hypothesised that the 3D conditions (automatic and interactive) will yield more symbolic responding and label generalisation in the ASC group compared to the 2D condition due to increased iconicity provided by the rotation (three-dimensional context), with greater label retention after a delay. Following the findings of previous research (Hartley and Allen 2015b) it is hypothesised that symbolic responding and label retention in the TD group will not differ between conditions. As interactivity has been found to complement the preferred learning style of children (Highfield and Goodwin 2013), it is hypothesised that both populations in the interactive condition will exhibit greater on-task engagement for both engagement measures (visual attention and communication) compared to the 2D and automatic conditions. Finally, based on previous research we expect greater engagement to be contingent symbolic understanding and label retention after a delay (Kucirkova 2014; Radesky et al. 2015; Xie et al. 2018).

## Method

### Participants

Ninety-six participants (34 female) were recruited for this study. There were 48 children with ASC (13 female) whose ages ranged from 4 years 11 months to 14 years 7 months (*M*_*age*_ = 9 years 0 months, *SD*_*age*_ = 23.12 months). They were recruited from five schools from North Wales and the north west of England and had been assessed by a qualified psychologist using standardised measures (ADOS, ADI-R), subsequently receiving a clinical diagnosis of autism. We further screened for the presence of symptoms using the current version of the Social Communication Questionnaire (SCQ; Rutter et al. 2003) completed by the class teacher (*M*_*score*_ = 19.33; *SD*_score_ = 6.18; range = 10–32).^2^ A further questionnaire was administered to class teachers to examine the use of The Picture Exchange Communication System and iPads/tablets in the classroom. The Picture Exchange Communication System was used as a language support by 38.1% of children (although PECS was not used during the task), and 88.1% used iPads/tablets at school (see Table 1 for frequency of iPad use for TD and ASC participants). Forty-eight TD children (21 female) also participated in the study, with ages ranging from 1 year 8 months to 6 years 9 months (*M*_*age*_ = 3 years 5 months, *SD*_*age*_ = 14.23 months). They were recruited from two nursery schools and one primary school in the North Wales area and 35.4% used iPads/tablets at school. As shown in Table 1, children with ASC were more frequent users of iPads or touch-screen devices (once a week or more) in school, χ^*2*^(1, *N* = 90) = 25.90, *p* < 0.001. As the experiment is a test of label mapping and retention, ASC and TD participants were matched for comparable levels of receptive vocabulary prior to the experimental tasks (see Table 2 for receptive vocabulary and non-verbal ability raw scores to enable comparison between groups). Due to behavioural difficulties (fussiness and inability to focus on the task), five children with ASC could not complete the training phase and were subsequently excluded from the study. Additional participants were recruited to ensure a total of 48 ASC children. All 48 TD children successfully completed the training phase and were included in the study.

**Table 1:** The percentages (and frequencies) of iPad/tablet use in school/nursery for ASC and TD participants

	ASC	TD
Question: Do children have experience with iPads or touch-screen devices in the nursery/in school?		
Every day	28.6% (12)	8.3% (4)
3–4 times a week	9.5% (4)	0% (0)
1–2 times a week	50.0% (21)	27.1% (13)
Not anymore but has in the past	11.9% (5)	0% (0)
Never	0% (0)	64.6% (31)

**Table 2:** The, mean (M), standard deviation (SD), range and number (N) of raw scores of participants for the British Picture Vocabulary Scale third edition (BPVS3—receptive vocabulary), Raven’s coloured progressive matrices (CPM—non-verbal IQ), the Wechsler Preschool and Primary Scale of intelligence third edition (WPPSI 3—non-verbal IQ) and chronological age

	ASC			*N*	TD			*N*	*p*
	*M*	SD	Range		*M*	SD	Range		
BPVS3	47.85	28.15	3–109	48	42.92	27.81	5–104	48	.39
CPM	19.27	8.61	7–31	22	23.25	7.41	17–33	4	
WPPSI 3	10.15	7.74	1–28	26	12.57	7.24	1–26	44	
Age	108.40	23.12	59–175	48	41.21	14.23	20–81	48	

Receptive vocabulary was measured using the British Picture Vocabulary Scale-3 (BPVS-3; Dunn and Dunn 2009). We report the raw scores as, for some participants, raw scores were too low to calculate a standardised score. The mean receptive vocabulary raw score for the BPVS-3 was 47.85 (range = 3–109) in the ASC group and 42.92 (range = 5–104) for the TD group, a non-significant difference, *t*(94) = − 0.86, *p* = 0.39, *d* = 0.18. The standardised scores for the TD group were all within an age-appropriate range. To further characterise the sample (although not for matching purposes), the Raven’s Coloured Progressive Matrices (CPM; Raven 2003) or the Block Design task of the Wechsler Preschool and Primary Scale of Intelligence—third edition (WPPSI-3; Wechsler 2002) were administered to participants as a measure of non-verbal ability. Twenty-two children with ASC (45.83%) and 4 children with TD (8.33%) over the age of 6, the minimum age suggested as appropriate for the test, completed the CPM. Twenty-six children with ASC (54.17%) who could not complete the CPM due to difficulty and 44 TD children (91.67%) below the age of 6 were assessed instead with the WPPSI-3. Although expressive vocabulary was not measured in this study, no non-verbal children were included in the study as confirmed by the class teacher.

### Materials

A 32 GB iPad Air 2 was used to present visual stimuli to participants in the training phase. Six unfamiliar objects were used in the study—consisting of a mixture of rubber dog toys and unusual household objects. Of the six unfamiliar objects, two were named target objects, two were unnamed distractor objects, and two were not shown on the iPad and were used only as distractor objects in the retention test. Although the two target objects were both dog toys, they differed in size, shape, colour and texture. No children expressed familiarity with the unfamiliar objects. Eight familiar objects were also shown on the iPad as distractor images, with four shown in each training phase. All familiar objects were selected from the Oxford CDI to ensure familiarity for children over 11 months of age. Stimuli were presented via an application developed for this study by a computer scientist at Lancaster University. This application facilitated presentation of real-world stimuli scanned into Object files (OBJ files) via a HP Sprout Pro 3D object scanning device. The application allowed for the images to be presented in each of the three conditions: 2D static presentation of images; automatic 360° rotation of the 3D image; and manual touch-screen 3D rotation, which was controlled by the participant. Images were presented for a duration of 6 s each, regardless of condition.

### Experimental Design

A between-subjects design with 3 conditions (2D, 3D automatic rotation and 3D interactive rotation) was used, with 16 participants from each group (ASC and TD) in each condition. Participants were assigned to conditions based on their BPVS scores, ensuring that there was a range of abilities in each condition and that there was no significant difference in receptive vocabulary score between conditions for the TD group, *F*(2,45) = 0.06, *p* = 0.95, *η*^2^ = 0.003, and the ASC group, *F*(2,45) = 0.27, *p* = 0.76, *η*^2^ = 0.01 (see Table 3).

**Table 3:** The, mean (M), standard deviation (SD), range and number (N) of raw scores of participants for the BPVS3 (receptive vocabulary measure) and chronological age across conditions for each group

	ASC			*N*	TD			*N*	*p*
	*M*	SD	Range		*M*	SD	Range		
BPVS3									
2D	43.69	31.60	3–109	16	44.56	28.86	5–104	16	.62
Automatic	48.94	30.80	4–104	16	41.19	29.98	8–98	16	.70
Interactive	50.94	23.44	22–99	16	43.00	26.19	8–92	16	.48
Chronological age									
2D	111.94	33.86	59–175		41.69	14.20	20–80	16	< .001
Automatic	106.81	14.77	76–133		39.25	14.48	20–81	16	< .001
Interactive	106.44	17.04	77–137		42.69	14.72	25–76	16	< .001

Counterbalancing was used to control for order effects. This included which target object (“Blicket” or “Toma”) was presented first in the mapping and generalisation tests, which target object was labelled “Blicket” and which was labelled “Toma”, the order of the stimuli in the mapping and generalisation tests, whether pictures or objects were shown first in the retention test and the order of stimuli presentation in the retention test.

### Procedure

Testing took place individually over three separate days. The first 2 days of testing were consecutive, followed by a 2-week gap before a test of retention. On the first day, participants were administered receptive vocabulary and cognitive measures. On the second day, participants were taken individually to the testing room, seated at a table adjacent to the experimenter and told that they were going to play a game on the iPad. A Samsung camcorder on a tripod was used to film the training phase and allowed for the coding of engagement. The camcorder was pointed towards the participant to allow for a clear view of the face and table-top. Participants completed 2 trials each comprising a training phase and the mapping and generalisation tests, thus in total there were two separate training phases, mapping tests and generalisation tests. The trials were separated by a 5-min break.

#### Training Phase

**Fig. 1:**
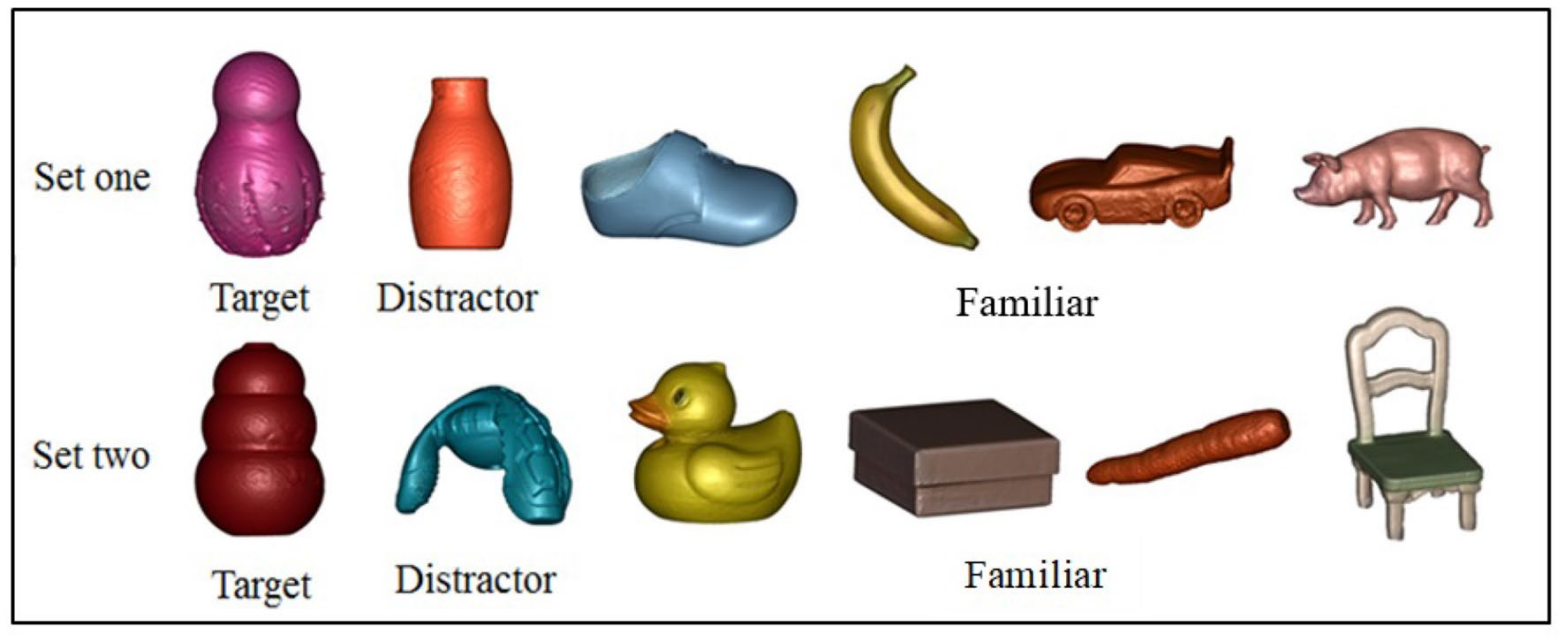
The two sets of stimuli presented to participants on the iPad in the training phase

To begin the training phase, the experimenter selected either the 2D, 3D automatic or 3D interactive condition on the iPad, as appropriate to the condition assigned to that child. The target image was presented four times within a sequence that consisted of an unfamiliar distractor image (also repeated four times) and four individual familiar images (shown once each), with the images presented in a fixed order (to ensure order was controlled across conditions), with the participants viewing a total of 12 images in the training phase, with each training phase lasting 72 s. The fixed order consisted of the target image first, followed by the distractor image and then the familiar image. The target image was labelled aloud by the experimenter on each instance of presentation with the unfamiliar label “this is a Blicket/Toma.” This label was repeated twice on each instance, as per previous research (Allen et al. 2015), giving a total of eight label repetitions per trial to maximise exposure to the novel label in a short time frame. This is because studies suggest that, despite a high level of accuracy with immediate fast-mapping of new words (Swingley 2010), successful label retention requires multiple instances of repetition (Axelsson and Horst 2014). Moreover, children with ASC may require multiple instances of labelling to learn a novel word due to difficulties in consolidating new word information (Haebig et al. 2017). The distractor object was accompanied with the verbal prompt “look at this.” The familiar objects were not labelled in the training phase and were not present in the mapping and generalisation tests. Figure 1 shows the two sets of images, for the two trials.

#### Mapping Test

**Fig. 2:**
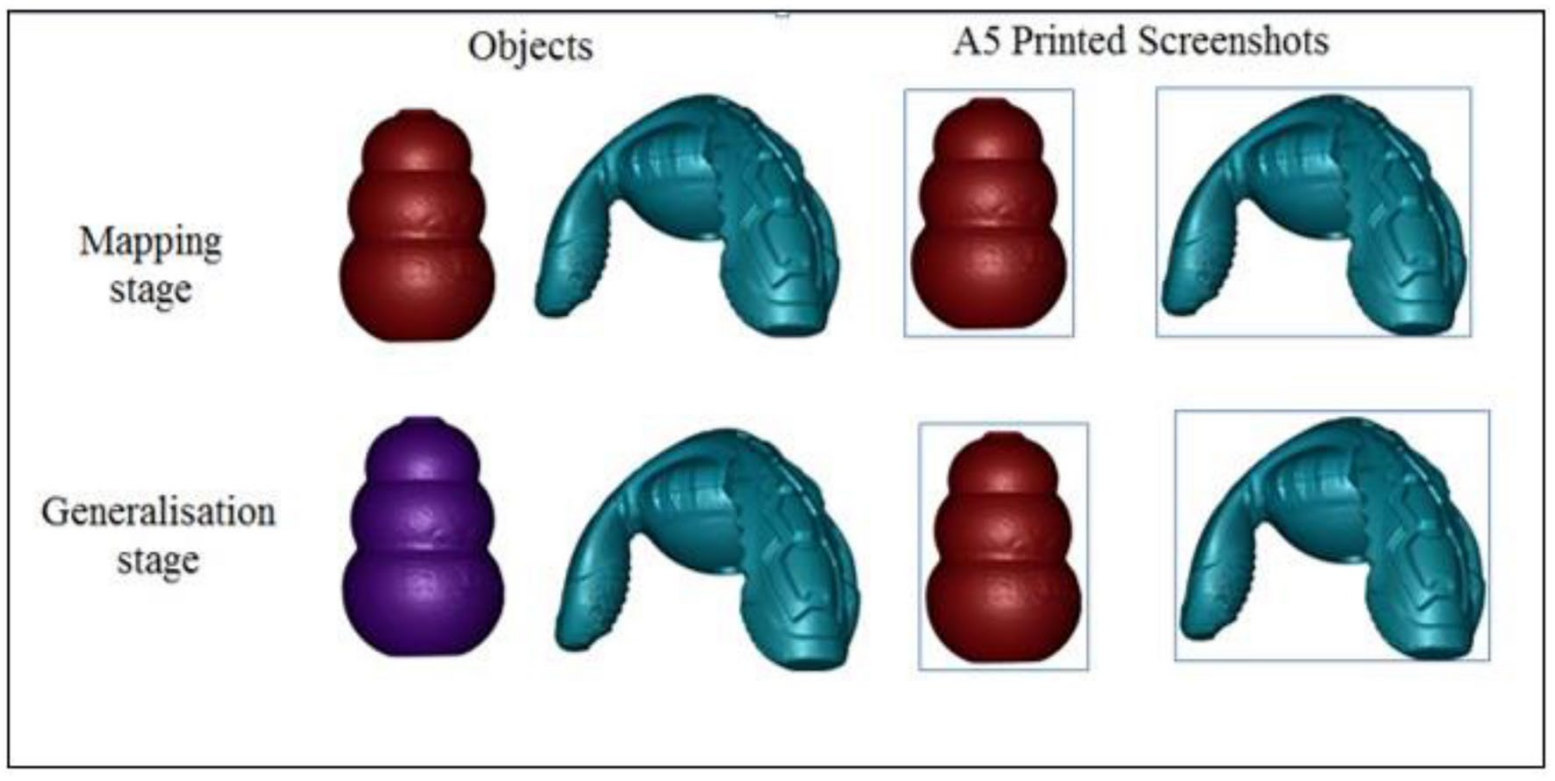
Example of array for mapping and generalisation tests for one target object

Following the training phase, participants completed a mapping test, designed to assess their symbolic understanding. They were shown an array of stimuli in a row in front of them, consisting of an A5 printed screenshot of the target object, an A5 printed screenshot of the distractor object, the target object in the original colour and the distractor object (see example in Fig. 2). Participants were then asked to identify the named object, with the experimenter requesting “show me a Blicket/Toma.” If the child had not understood the referential function of the image in the training phase, it was expected that they would only select the target image, thereby restricting the label to the picture itself. If the child had understood the referential nature of the image in the training phase, it was expected that they would select the target object or both the target image and target object, generalising the label from the picture to its real-world referent and taken as a measure of symbolic understanding (see Allen et al. 2015).

#### Generalisation Test

After the mapping test, participants completed the generalisation test in which they were shown an array of stimuli in a row consisting of the same stimuli as the mapping test but with a differently coloured version of the same target object. Participants were asked again to “show me a Blicket/Toma.” Children who had not formed a referential understanding of the image and had selected the target picture alone in the mapping test were expected to do so again in the generalisation test. As some children with ASC have specific difficulties generalising a novel label from the original exemplar to a differently coloured version, it was expected that some children in the ASC sample would select the target picture alone in the generalisation test despite selecting the object in the mapping test. In each stage of the experiment, positive reinforcement was given only to reinforce good behaviour and attention and was not directed towards task performance.

#### Retention Test

Participants were tested approximately two weeks later (*M*_*days*_ = 16.31, *SD*_*days*_ = 2.62) to examine word–picture–referent mapping after a delay. Participants were shown an array of stimuli in front of them, consisting of a total of three A5 pictures, one of the first target object, a novel distractor from the immediate recall test and a new novel distractor, shown in a counterbalanced order. They were asked “show me the Blicket/Toma.” This was then repeated with the actual objects instead of pictures and participants were again asked “show me the Blicket/Toma.” Both tasks were then repeated for the second target object.

### Data Coding

#### Training Phase

All videos of the training phase were analysed for participant engagement by two independent coders, who analysed each entire video. Participant engagement was divided into two categories with individual sub-categories (see Table 4). As per Richter and Courage (2017), visual attention (towards the screen, adult or environment) was coded based on looking duration (in seconds). Communication (relevant speech and labelling) was coded on each instance. The duration of looks towards each sub-category was measured using the time data displayed on the video, and the total time for each sub-category was summed once coding was completed. For communication, each instance of relevant speech and labelling was coded and again an overall total was created for each sub-category. It is important to note that the video-coders did not define individual participants as “engaged” or “disengaged” based on their engagement scores. Instead, more visual attention and instances of communication in certain categories provided an indication of degree of engagement with the task (total looking time at the screen, relevant speech and labelling), while others provided an indication of the extent of social engagement (adult-oriented looking time) and task disengagement (off-screen (environment) looking time).

**Table 4:** The description and maximum possible scores and inter-rater reliability of the two engagement categories and their sub-categories

Engagement category	Sub-category	Description	Maximum score	Inter-rater reliability
Visual attention	Total screen looking time	Total amount of time the participant looks the screen. Greater total screen looking time would here indicate greater task engagement	72 s	.97
	Adult-oriented looking time	Total amount of time the participant looks at the adult. Greater adult-oriented looking time would here indicate greater social engagement	72 s	.97
	Off-screen (environment) looking time	Total amount of time the participant looks away from the screen (excluding looking time at the adult). Greater off-screen (environment) looking time would here indicate greater disengagement with the task	72 s	.97
Communication	Relevant speech	Total instances of speech (word, phrase or sentence—each defined as one instance) relevant to the task or the images on the screen (excluding labelling the target image). More instances of relevant speech would here indicate greater task engagement E.g. “Oh look, another one!” E.g. “Duck!”	No maximum	.98
	Labelling	Whether or not the participant labels the target image for each individual instance of presentation. More instances of labelling would here indicate greater task engagement	4 instances of labelling—whether or not they label each of the 4 target images per trial	.98

An intra-class correlational analysis with fixed effects and absolute agreement was conducted between the primary and secondary video-coder for each sub-category separately and all ratings were found to be greater than 0.97 (see Table 4 for reliability ratings for each sub-category). This represents high agreement according to Cicchetti (1994) where scores on or above 0.75 are classified as ‘excellent’. Therefore, the primary video-coder’s scores were used for analysis. Engagement scores were averaged across trials to create one total score for each participant.

#### Mapping and Generalisation Tests

Item selection was coded by the researcher during the experiment (as per Allen et al. 2015; Hartley and Allen 2015b). Item selection was defined as the child clearly pointing to particular items in the array or handing items to the experimenter in response to the question “Show me a Blicket/Toma.” Only explicit responses were coded (pointing, giving or sliding the item towards the experimenter) as per Preissler (2008).

#### Consistent Symbol Mapping Across Trials

We were interested to see whether participants showed consistent responding across trials (see Joseph et al. 2019); in this way we could classify children as consistent symbolic responders or not across both mapping and generalisation trials. We defined consistent symbolic responding as a selection of the target object with or without the target picture in mapping tests (trial one and two), and also across generalisation tests (trial one and two). All other combinations of responses (associative responding, selecting distractor items, and symbolic responding on one trial only) were categorised as “not consistent.” Binary logistic regressions were conducted for “consistent” and “not consistent” responses for mapping tests and generalisation tests separately. We then coded responses across mapping and generalisation tests to determine how robust children’s responses were: Participants were categorised as “robust symbolic” when they demonstrated symbolic responding (selecting the target object with or without the target picture) across all tests (mapping and generalisation) for both trials. All other combinations of responses (associative responding, selecting distractor items and inconsistent symbolic responding) were categorised as “not robust.”

## Results

We first analysed results of the mapping and generalisation tests separately, then looked at how individuals performed across both mapping and generalisation tests together. We then assessed whether children retained the new labels after a 2-week delay. Finally, we evaluated levels of engagement during the training phase, and determined whether this related to performance.

### Mapping Tests Combined

Table 5 shows individual responses in the mapping tests. To check for consistency of responses, we combined the two trials. 68.8% of ASC participants and 60.4% of TD participants demonstrated consistent symbolic responding across both mapping tests. A binary logistic regression found no significant association between consistency of symbolic responding and group and condition, χ^*2*^(3) = 1.10, *p* = 0.77. There was no significant interaction between group and condition, χ^*2*^(2) = 0.09, *p* = 0.96.

**Table 5:** The number and percentage of participant responses for mapping tests one and two

ASC				TD			
Response	2D (%)	Automatic (%)	Interactive (%)	Response	2D (%)	Automatic (%)	Interactive (%)
Trial one mapping test							
Picture	1 (6.3)	1 (6.3)	0 (0.0)	Picture	1 (6.3)	0 (0.0)	2 (12.5)
Object	13 (81.3)	12 (75.0)	5 (31.3)	Object	8 (50.0)	14 (87.5)	7 (43.8)
Both	1 (6.3)	1 (6.3)	7 (43.8)	Both	5 (31.3)	2 (12.5)	4 (25.0)
Distractor	1 (6.3)	2 (12.5)	4 (25.0)	Distractor	2 (12.5)	0 (0.0)	3 (18.8)
Trial two mapping test							
Picture	0 (0.0)	1 (6.3)	0 (0.0)	Picture	1 (6.3)	4 (25.0)	1 (6.3)
Object	8 (50.0)	11 (68.8)	8 (50.0)	Object	7 (43.8)	9 (56.3)	7 (43.8)
Both	3 (18.8)	2 (12.5)	6 (37.5)	Both	4 (25.0)	1 (6.3)	4 (25.0)
Distractor	5 (31.3)	2 (12.5)	2 (12.5)	Distractor	4 (25.0)	2 (15.4)	4 (25.0)

### Generalisation Tests Combined

60.4% of ASC participants and 58.3% of TD participants demonstrated consistent symbolic responding across both generalisation trials. A binary logistic regression found no significant association between consistency of symbolic responding and group and condition, χ^*2*^(3) = 1.85, *p* = 0.60. There was no significant interaction between group and condition, χ^*2*^(2) = 2.25, *p* = 0.33 (see Table 6 for all responses in the generalisation tests).

**Table 6:** The number and percentage of participant responses for generalisation tests one and two

ASC				TD			
Response	2D (%)	Automatic (%)	Interactive (%)	Response	2D (%)	Automatic (%)	Interactive (%)
Trial one generalisation test							
Picture	3 (18.8)	1 (6.3)	0 (0.0)	Picture	3 (18.8)	2 (12.5)	2 (12.5)
Object	9 (56.3)	11 (68.8)	5 (31.3)	Object	4 (25.0)	11 (68.8)	8 (50.0)
Both	2 (12.5)	0 (0.0)	6 (37.5)	Both	5 (31.3)	3 (18.8)	4 (25.0)
Distractor	2 (12.5)	4 (25.0)	5 (31.3)	Distractor	4 (25.0)	0 (0.0)	2 (12.5)
Trial two generalisation test							
Picture	0 (0.0)	1 (6.3)	0 (0.0)	Picture	1 (6.3)	1 (6.3)	2 (12.5)
Object	10 (62.5)	12 (75.0)	9 (56.3)	Object	9 (56.3)	8 (50.0)	8 (50.0)
Both	1 (6.3)	1 (6.3)	5 (31.3)	Both	4 (25.0)	4 (25.0)	3 (18.8)
Distractor	5 (31.3)	2 (12.5)	2 (12.5)	Distractor	2 (12.5)	3 (18.8)	3 (18.8)

### Robust Symbol Mapping Across Trials

Here, we investigated response patterns across mapping and generalisation tests when taken together by examining whether or not participants were “robust symbolic” responders. 54.2% of ASC participants and 47.9% of TD participants were robust across both trials. A binary logistic regression found no significant association between robust symbolic responding and group and condition, χ^*2*^(3) = 2.73, *p* = 0.44. There was no significant interaction between group and condition, χ^*2*^(2) = 1.47, *p* = 0.48 (see Table 7 for all scores).

**Table 7:** The number (and percentages) of robust symbolic responding (robust and not robust) across all test trials and the mean (and standard deviation) of labels correctly assigned to their target pictures/objects in the retention test

	ASC			TD		
	2D	Automatic	Interactive	2D	Automatic	Interactive
Robust (%)	7 (43.8)	10 (62.5)	9 (56.3)	6 (37.5)	9 (56.3)	8 (50.0)
Not Robust (%)	9 (56.3)	6 (37.5)	7 (43.8)	10 (62.5)	7 (43.8)	8 (50.0)
Number of labels	2.20 (1.74)	2.77 (1.42)	2.07 (1.44)	3.00 (1.46)	2.75 (1.44)	1.80 (1.82)

### Retention Test

Due to school absences, only 90 out of 96 participants (93.75%) completed the retention test. Five children with ASC (10.4%) and 1 TD child (2.1%) did not complete the retention test. Out of a total of 4 possible instances of labelling in the retention test—trial one (picture and object) and trial two (picture and object)—participants correctly assigned a mean of 2.43 labels (*SD* = 1.58) to their target images/objects (see Table 7). No significant difference in retention was found for group, *F*(1,84) = 0.27, *p* = 0.61, *η*^*2*^ = 0.003, or condition, *F*(2,84) = 2.34, *p* = 0.10, *η*^2^ = 0.05 and no significant interaction was found between group and condition, *F*(2,84) = 0.97, *p* = 0.38, *η*^2^ = 0.02.

### Participant Engagement Coding

Time data were analysed for the visual attention measures and frequency data were analysed for communication. Individual participant data from both trials were averaged to create a combined total score for each measure (see Table 8 for all engagement scores).

**Table 8:** Mean (and standard deviation) of engagement scores averaged across trials one and two

	ASC			TD			Sig group differences
	2D	Automatic	Interactive	2D	Automatic	Interactive	
Visual attention							
Total screen looking	55.53* (14.55)	58.11* (9.98)	68.25* (5.76)	52.80* (12.85)	59.11* (10.63)	65.00* (8.30)	*p* = .01
Adult-oriented looking	3.41* (2.78)	5.00 (5.79)	0.75* (1.02)	11.30* (10.07)	6.39 (4.64)	3.63* (6.44)	*p* < .001
Off-screen (environment) looking	13.06* (14.58)	8.89 (11.54)	3.00* (5.56)	7.90* (8.04)	6.50 (6.95)	3.37* (4.29)	*p* = .22
Communication							
Relevant speech	4.19* (3.90)	4.57* (4.96)	2.00* (2.48)	6.83* (3.34)	5.57* (4.60)	3.37* (3.14)	*p* = .04
Labelling	1.22* (1.15)	1.29 (1.42)	0.81* (0.89)	2.20* (1.41)	1.07 (1.30)	0.40* (0.83)	*p* = .64

All looking times are calculated in seconds. Speech is calculated in instances

^*^Represents p < .05. Differences are reported for group and condition

### Visual Attention

Looking time proportions between the screen, adult and off-screen (environment) indicated a high level of engagement in the task for both groups. Children with ASC spent 84.4% of time looking at the screen compared to 4.1% looking towards the adult and 11.5% looking off-screen (environment). TD children spent 81.9% of time looking at the screen compared to 9.9% looking towards the adult and 8.1% looking off-screen.

Total screen looking time was analysed using a two-way ANOVA with group and condition as factors. No difference was found between groups, *F*(1,84) = 0.54, *p* = 0.47, *η*^2^ = 0.01. A main effect of condition was found, *F*(2,84) = 10.66, *p* < 0.001, *η*^2^ = 0.20. Tukey post-hoc analysis showed significantly higher total screen looking time in the interactive condition (*M* = 66.68 s) compared to the 2D condition (*M* = 54.21 s) and the automatic condition (*M* = 58.61 s). No significant interaction was found between group and condition, *F*(2,84) = 0.34, *p* = 0.72, *η*^2^ = 0.01.

Off-screen looking time was split into adult-oriented looking time and off-screen (environment) looking time. As these measures are mutually exclusive, only adult-oriented looking time is reported here. These data were analysed using a two-way ANOVA with group and condition as factors. A main effect of group was found, *F*(1,84) = 10.89, *p* = 0.001, *η*^2^ = 0.12. The TD group looked significantly longer at the adult (*M* = 7.13 s) compared to the ASC group (*M* = 2.97 s). A main effect of condition was also found, *F*(2,84) = 6.33, *p* = 0.003, *η*^2^ = 0.13. Tukey post-hoc analysis showed significantly greater adult-oriented looking time in the 2D condition (*M* = 7.23 s) compared to the interactive condition (*M* = 2.15 s). No significant interaction was found between group and condition, *F*(2,84) = 2.57, *p* = 0.08, *η*^2^ = 0.06.

### Communication

On average, children with ASC produced 3.54 instances of relevant speech per trial and TD children produced 5.25 instances of relevant speech per trial (see Table 8). Relevant speech was analysed using a two-way ANOVA with group and condition as factors. A main effect of group was found, *F*(1,84) = 4.35, *p* = 0.04, *η*^2^ = 0.05. The TD group produced significantly more instances of relevant speech (*M* = 5.25 instances) than the ASC group (*M* = 3.54 instances). A main effect of condition was also found, *F*(2,84) = 4.93, *p* = 0.01, *η*^2^ = 0.11. Tukey post-hoc analysis found significantly more instances of relevant speech in the 2D condition (*M* = 5.47 instances) and the automatic condition (*M* = 5.07 instances) compared to the interactive condition (*M* = 2.66 instances). No significant interaction was found between group and condition, *F*(2,84) = 0.39, *p* = 0.68, *η*^2^ = 0.01.

On average, children with ASC produced 1.10 out of 4 possible instances of labelling and TD children produced 1.23 out of 4 possible instances of labelling of the target image per trial (see Table 8). Labelling was analysed using a two-way ANOVA with group and condition as factors. No significant group difference was found, *F*(1,84) = 0.22, *p* = 0.64, *η*^*2*^ = 0.003. A main effect of condition was found, *F*(2,84) = 6.73, *p* = 0.002, *η*^*2*^ = 0.14. Tukey post-hoc analysis found significantly more instances of labelling in the 2D condition (*M* = 1.69 instances) compared to the interactive condition (*M* = 0.61 instances). No significant interaction was found between group and condition, *F*(2,84) = 3.11, *p* = 0.05, *η*^*2*^ = 0.07.

### Engagement and Performance

In this section, we examine whether there is a relation between engagement (screen looking time) and symbolic responding and label retention (both immediate and in the retention test) for the ASC and TD groups respectively. Although group differences did not emerge in our earlier analyses, the literature and our earlier predictions suggested that different factors might underlie performance (Field et al. 2016a).

### Robust Symbolic Responding

An independent samples t test was conducted to examine whether engagement and immediate robust symbolic responding differed between groups. For the ASC group, a significant difference in engagement was found between robust and non-robust symbolic responders, *t*(44) = − 2.49, *p* = 0.02, *d* = 0.76. Robust symbolic responders had greater screen looking time (*M* = 64.72 s) than non-robust symbolic responders (*M* = 56.00 s). No significant difference was found for the TD group, *t*(42) = − 1.42, *p* = 0.16, *d* = 0.43. We also wanted to check whether robust symbolic performance was related to PECS useage for the ASC group. We found a significant  negative correlation between PECS use and robust symbolic performance, *r* = − 0.39,* p* = 0.01.

### Retention Test

An independent samples t-test was conducted to examine whether performance on the retention test (time 2) differed between robust and non-robust responders at time one. For the ASC group, a significant difference in retention was found between robust and non-robust symbolic responders, *t*(41) = − 2.18, *p* = 0.04, *d* = 0.66. Robust symbolic responders scored higher on the retention test (*M* = 2.78) than the non-robust symbolic responders (*M* = 1.80). No significant difference was found for the TD group, *t*(45) = − 1.22, *p* = 0.23, *d* = 0.36.

A correlation was conducted to examine the relationship between screen-looking time and performance on the retention test for both groups. No significant relationship was found for the ASC group, *r* = − 0.01, *n* = 42, *p* = 0.94, or the TD group, *r* = 0.01, *n* = 44, *p* = 0.98.

## Discussion

This study investigated whether symbolic responding and label retention differ between children with ASC and TD children when given a new label for novel “three-dimensional” images (either automatically rotating or interactive) compared to 2D static images on an iPad screen. Contrary to predictions, we did not find any group or condition differences: both groups demonstrated a similar level of symbolic understanding and label retention across the three different presentation conditions. We found similar levels of on-screen attention to the task in both groups, but different patterns of task performance emerged. We discuss these findings in turn.

As expected, we found no difference in symbolic responding and label retention amongst conditions for the TD group. However, contrary to our hypothesis, we also found no difference in performance between conditions for children with ASC. These results suggest that enhancing iconicity through motion and interactivity does not increase symbolic understanding over and above static, non-interactive, coloured stimuli. One possible explanation for the absence of an effect is that motion and interactivity may impede dual representation (Uttal et al. 1998). Dual representation is the understanding that a symbol can be both an object in its own right while also representing something else in the environment, such as an image being both a picture and also a symbol for a real-world referent (DeLoache 1987, 1991, 1995). Increasing the interest and attractiveness of a symbol can make it difficult for children to think of a symbol both referentially and as a concrete object (DeLoache 2004; Uttal et al. 1998), potentially masking any potential gains that might be achieved by increasing perceptual iconicity.

Collectively, the results show variation in performance for both the ASC and TD samples, with only half of the cohort reliably symbolic. Two explanations may account for such variation. One possibility concerns our relatively strict coding: children needed to demonstrate symbolic understanding on all 4 trials to be considered ‘robust’. This is different from past research that consisted of single trials and forced choice responses between the picture and object in the absence of distractors (Hartley and Allen 2015b; Preissler 2008). Thus, our study provided more opportunity for error, but also provides a more stringent test of symbolic understanding. A second explanation is that the acquisition of symbolic understanding is not a qualitative step-change in a Piagetian sense (Fischer and Silvern 1985; Piaget 1936), but something that develops over time and varies with input and experience. Children may also be testing out various strategies (Alibali 1999), in which they switch to more accurate and efficient methods of learning (Siegler 2006).

Despite the variation in overall robust symbolic responding, very few ‘associative’ responses were made, even in the 2D condition for either population. It is possible that there is a threshold over and above which any enhancement to iconicity will not benefit performance. Perhaps colour photographs, already considered to be ‘transparent’ symbols, are enough to promote symbolic understanding (see also Hartley and Allen 2014b). Indeed, our baseline level of iconicity was colour photographs, in contrast to previous research which has included symbols with lower iconicity such as black and white line drawings and cartoons. This may explain why we found only an average of 3.1% associative responses across trials, compared to prior studies using a similar design (55% in Preissler (2008) and an average of 62.9% in Hartley and Allen (2015b)). Hartley and Allen (2015b) found a large difference between black and white line drawings and coloured photographs, with associative responding decreasing by 25% when colour photographs were used. We thus appear to have provided optimal conditions for word–picture–referent mapping in the current study and it is encouraging that under such conditions our ASC group were just as successful as their TD peers.

As predicted, and in line with Richter and Courage (2017), the interactive condition increased the visual attention (e.g. on screen looking) of both groups. However, instances of communication (relevant speech and labelling) decreased for both groups in the interactive condition compared to the 2D condition. These results suggest that interactive stimuli increase engagement in terms of looking behaviour but may decrease social communication. It is possible that on-screen interactivity can either be beneficial or detrimental to engagement depending on the specific needs of the learner. To facilitate focus on a task, an interactive application may serve to increase attention and prevent external distraction which could inhibit learning (Oakes et al. 2004). However, iPad applications may not be the optimal method to foster social communication and engagement between the teacher and the learner, a skill that is typically dimished in children with ASC (Wodka et al. 2013). Although the adult provided a degree of mediation through co-viewing in all conditions, it is possible that interactive features may reduce the opportunities for active adult mediation—such as responding to participant comments and questions—as children are distracted with their individual touch-screen exploration (Nathanson 2001). Instances of relevant speech in the ASC group dropped by 50% in the interactive condition. Moreover, adult oriented looking time reduced by 85% between the automatic condition and the interactive condition. This suggests that physical manipulation of stimuli reduces spontaneous communication and social interaction compared to passively viewing stimuli in this population. Previous research has found that touch-screen interventions for social communication do not transfer into real-world communication skills (Fletcher-Watson et al. 2016), despite high engagement in the task. It is possible that the self-contained nature of learning through this medium (Allen et al. 2016) and the increased cognitive load provided by interactive touch-screen features (Kirkorian 2018; Richter and Courage 2017) may diminish the need to share salient information with the adult and may be a particular hinderance to the facilitation of social interaction in individuals with ASC. Non-interactive presentation of learning material may be optimal for increasing social communication in this population.

A different pattern of looking time was observed for the ASC group compared to the TD group. Despite similar proportions of on-screen looking time, the ASC group predictably looked less at the adult (Constantino et al. 2017; Jones et al. 2008; Kasari and Patterson 2012). Moreover, as expected, the TD group was found to have significantly higher levels of relevant speech than the ASC group, in line with previous research (Anderson et al. 2007; Dawson et al. 2004; Wodka et al. 2013).

Finally, consistent with our hypothesis that engagement would be associated with performance, it was found that robust symbolic responders engaged in significantly more on-screen looking time than non-robust symbolic responders in the ASC group alone. This may be due to increased attention to relevant stimuli, preventing distractions which could impede task performance (Oakes et al. 2004), which is particularly important for children with poorer executive functioning (Richter and Courage 2017), such as those with ASC (Finnegan and Mazin 2016). However it is important to note that, while based on prior coding schemes (Richter and Courage), attention is multi-faceted and defining what is on-task behaviour is complex (Knudsen 2007). For example, although children may demonstrate a high level of visual attention towards the screen, we do not know precisely what they are attending to with observation alone. Future research could use eye-tracking to more accurately define whether participants are attending to on-task (target stimuli) or off-task (background) information.

Interestingly, screen looking time was not related to task performance on the follow-up test of label retention two weeks later. Instead, robust symbolic responders at time one had significantly greater retention for the ASC group only, with no significant difference found for the TD group. It appears that whether children treat pictorial symbols as referential (i.e. symbolic) has an impact on their subsequent retention of a new label. Future research should investigate whether this specifically affects encoding or retrieval processes (Bowler et al 2004; Ben-Shalom 2003).

## Limitations

In addition to the limitations discussed above, we detail here the four most pertinent for future research. First, a potential explanation for the comparable levels of symbolic understanding between ASC and TD groups in this study may be that our ASC group had a lower mean SCQ score by 8.17 points compared to previous research (Allen et al. 2015). This suggests that the current sample consists of higher-functioning ASC participants than past studies; it is possible that minimally verbal children with ASC are more natural associative learners (Preissler 2008) and that the heterogeneity of the condition and language profile (Allen and Yau 2019) implicates different routes of learning word–picture–object relations across individuals with ASC. To investigate this further, future research should compare ASC participants with range of abilities, such as lower-functioning/minimally verbal children with ASC with higher-functioning/verbal ASC participants using the same methodology.

Second, children were matched on their receptive vocabulary score and, as per previous research, were not matched on chronological age (Field et al. 2016; Maljaars et al. 2012; Tager-Flusberg 1985; Tek et al. 2008). Children with ASC are a heterogenous population in which overall receptive language ability and functioning can vary significantly despite chronological age (Weismer et al. 2010). Thus, to match for chronological age would most likely have resulted in a control sample with higher verbal skills that fell into a narrower range of performance. However, we acknowledge that age is a good proxy for increasing vocabulary ability in TD populations (Dunn and Dunn 2009) and may influence performance. It is also important to note that, as the BPVS3 provides an age-equivalent score from 45 months and over, some of our participants could not be provided with an age-equivalent score as they were either too young (in the TD group) or scored too low (in the ASC group). However, as it was crucial for children to be matched on their receptive vocabulary, as this is a task of label mapping and retention, children were matched on raw scores in this study.

Third, although our study goes beyond the single trial methodology of previous research, two trials still cannot be generalised to symbol learning at large. Future research should increase the number of trials to increase the generalisability of findings to real-world symbol learning. Finally, word–symbol–referent mapping studies to date have focussed on the teaching of new noun labels (Allen et al. 2015; Hartley and Allen 2015b; Preissler and Carey 2004; Preissler 2008). However, in order to be representative of word acquisition as a whole, the symbolic mapping of other word-types (such as adjectives and verbs) should be examined in future work in this area.

## Conclusion

Overall, this study suggests that children with ASC are just as able as vocabulary-matched peers to treat pictures symbolically and retain new labels at the same rate after a delay. Increasing the iconicity of pictures to a ‘transparent’ (Fuller et al. 1997) level through two-dimensional colour photographs may be sufficient to elicit the maximum benefit to symbolic understanding in ASC, evidenced by our lack of condition difference when rotation and interactivity were added to the task. However, interactivity has been found to increase engagement in terms of visual on-task attention for both groups, at the possible expense of communication. This finding may have important implications for learning through the medium of iPads/tablets, suggesting that iPads/tablets can be successful to elicit some skills (such as greater visual attention) and unsuccessful at eliciting others (such as social communication). These findings suggest that practitioners need to clarify their purpose—how and why—they use electronic education due to the different pattern of findings for word learning and engagement. Taken together, our results suggest that there is a link between engagement and task performance for individuals with ASC, and that different routes to symbolic understanding may be implicated in typical and atypical development.

## Footnotes

1 As ‘persistence, enthusiasm and compliance’ was measured through looking visual attention and communication, and so overlapped with the above engagement measures, this was removed as a distinct category.

2 38 participants scored 15 or above, the suggested cutoff for ASD. 6 participants scored between 12–14, and 4 participants scored below 12. As all of our participants had a clinical diagnosis of autism, and given the caution regarding false negatives obtained with the SCQ (Rutter et al. 2003), and suggestion that lower cutoffs are sometimes appropriate (Eaves et al. 2006; Norris and Lecavalier 2010) we included all participants in the analysis.
